# Digital inequality in Ecuador: a multivariate analysis of ICT access and use using multiple correspondence analysis (MCA) and ENEMDU 2025 data

**DOI:** 10.3389/fsoc.2026.1770327

**Published:** 2026-08-05

**Authors:** Dario Javier Cervantes Diaz, Edgar Rolando Morales Caluña

**Affiliations:** 1 Carrera de Derecho, Universidad Estatal de Milagro, Milagro, Ecuador; 2 Carrera de Nutrición y Dietética, Universidad Estatal de Milagro, Milagro, Ecuador

**Keywords:** digital inequality, Ecuador, ENEMDU 2025, ICT access, multiple correspondence analysis

## Abstract

**Background:**

Digital inequality remains a persistent challenge in middle-income countries, where access to information and communication technologies (ICTs), digital skills, and usage patterns are unevenly distributed across social groups. In Ecuador, despite policy efforts to expand connectivity, significant disparities persist across demographic and territorial dimensions.

**Methods:**

This study analyzed digital inequality using nationally representative data from the ICT module of the ENEMDU 2025 survey. A cross-sectional design was applied, incorporating both household and individual data. Descriptive statistics, chi-square tests, Cramér's V, and Multiple Correspondence Analysis (MCA) were used. All analyses were conducted in Python using Google Colab, applying expansion factors to ensure national representativeness.

**Results:**

Internet access reached 71.3% of households, while 82.4% of individuals reported internet use. However, marked inequalities were identified. Age emerged as the strongest determinant, with substantially lower usage among older adults. Significant urban-rural gaps were observed in both internet access and computer ownership. No statistically significant gender differences were found in mobile phone ownership. MCA revealed distinct profiles of digital inclusion and exclusion shaped by age, education, and territory.

**Discussion:**

Digital inequality in Ecuador is multidimensional and structurally embedded. Expanding connectivity alone is insufficient; targeted policies addressing digital skills, access to productive technologies, and territorial disparities are necessary to achieve inclusive digital development and reduce persistent inequalities.

## Introduction

Digital technologies have become central to economic participation, education, social interaction, and civic engagement. However, access to and effective use of digital resources remain unevenly distributed across social groups (ITU, 2023; Lybeck et al., 2024; OECD, 2023). Despite steady global growth in internet penetration, substantial disparities persist in connectivity, device ownership, digital skills, and meaningful use (Hollimon et al., 2025). These inequalities—conceptualized in the literature as first-, second-, and third-level digital divides—reflect and often reproduce broader socio-economic stratification. The problem is that a lot of people do not have access to the internet or know how to use it. This is an issue all around the world. In the year 2021 about 63% of people in the world were using the internet, which means a lot of people were still not connected to the internet and could not use all the things it has to offer. The internet is really important, for resources. People need resources and the internet to do well in life. Scholars note that simply expanding infrastructure is not enough to eliminate digital inequality, as structural differences in education, income, and social capital shape access, skills, and outcomes (Ragnedda et al., 2022; Scheerder et al., 2017; Szabó, 2024). In other words, digital exclusion becomes a self-reinforcing loop: individuals and households lacking affordable connectivity, diversified devices, and advanced digital skills fall further behind economically and socially (Bergantino et al., 2025; Ragnedda et al., 2022; van Deursen and van Dijk, 2019). Grinevich et al. (2025), for example, show that social and economic vulnerabilities tend to coincide with digital exclusion in a “self-reinforcing” way. This multidimensional view of the digital divide is increasingly recognized by international agencies (ITU, OECD, etc.), which emphasize that age, education, income, and geography correlate strongly with ICT access and use. In sum, at the global level there is broad consensus that advancing connectivity must go hand-in-hand with education, training, and inclusive policies to ensure that all segments of society can benefit from digital technologies (Magoutas et al., 2024).

Despite global progress, significant disparities persist across regions. In Latin America and the Caribbean (LAC), for instance, recent UN data highlight a persistent urban–rural divide: roughly 77% of urban households have Internet connectivity, whereas only 38% of rural households do (ECLAC, 2024). Wealth and social status also shape connectivity: in some LAC countries in 2022, Internet penetration in high-income homes was nearly double that in the poorest households. These regional gaps are symptomatic of broader structural inequalities. As a case in point, the *Economic Commission for Latin America and the Caribbean* (ECLAC) launched a Digital Development Observatory in early 2024 to track such divides and support policy—underscoring that, at present, the region lags far behind more developed areas (e.g., LAC fixed broadband penetration is below 20, vs ~40% in Europe; ECLAC, 2024). In Latin America's largest economies, targeted policies have begun to emerge: for example, Ecuador in 2017 passed legislation enabling social data tariffs to subsidize Internet service for vulnerable, low-income populations (Zaballos et al., 2024). Such measures reflect growing policy awareness of the digital divide, but persistent challenges remain, particularly in rural and disadvantaged urban areas. Ecuador's own experience illustrates these trends. The country has seen rapid digital growth—by 2024 it had over 15 million Internet users and a reported 83.7% internet penetration—but access is uneven (Digital Economy, 2025). According to the national telecom regulator, only about 74.4% of Ecuadorians had Internet access by end-2022 (up from 71.8% the year before), meaning roughly one-quarter of the population was still offline at that time. Access disparities are stark: broadband adoption reaches just ~20% in rural/remote communities vs. over 70% in major cities (Digital Economy, 2025). This digital gap mirrors traditional inequalities: for example, households headed by individuals with lower educational attainment or income levels lag significantly in Internet use (Scheerder et al., 2017). Recent analyses using Ecuadorian data confirm these patterns. Moreno-Vallejo et al. (2025) report that ICT usage grew across all age groups from 2023 to 2024, driven chiefly by education level (highest correlated factor), yet the urban–rural gap remained pronounced (Moreno-Vallejo et al., 2025). In parallel, Ecuador's government has launched a *Digital Transformation Agenda 2025–2030* (MINTEL, 2025) and other initiatives (e.g., public computer centers, ICT subsidies) to bolster connectivity and skills in underserved areas. Nonetheless, observers point out that “specificities in digital exclusion” still need to be addressed at both local and national governance levels in Ecuador and that successful e-inclusion strategies will require coordinated action across public, private and social sectors (De la Cruz Campos et al., 2024). To date, much of the research on Ecuador's digital divide has been descriptive or qualitative. A recent systematic review by De la Cruz Campos et al. (2024) confirms that Ecuadorian studies on digital inclusion (largely published 2014–2022) have identified the main causes of the divide (such as technology infrastructure deficits, education gaps, and rural isolation) and proposed broad solutions (like educational policies and new tech access points). However, fewer studies have performed detailed quantitative analyses of ICT access and use at the household or individual level, partly due to data limitations. The 2022 revival of Ecuador's ICT survey module offers new opportunities. Between 2008 and 2017 the Encuesta de Empleo, Desempleo y Subempleo (ENEMDU) periodically collected household ICT data, and from 2018 to 2020 a multipurpose survey carried similar modules. Recognizing the value of this information, Ecuador's National Statistics Institute (INEC, 2025) reintroduced an expanded ICT module into ENEMDU for 2022–2025. According to INEC, the module's aim is “to generate information on ICT equipment, and on household access to and use of the Internet and cellphones, providing inputs for analysis and public policy”. Indeed, the 2025 ENEMDU ICT results show that out of about 5.26 million surveyed households, 3.75 million had Internet access (≈71.3%), while 1.51 million (≈28.7%) did not—quantifying the gap that must be bridged. Similarly, among the 17.4 million Ecuadorians aged 5 and over, 13.9 million (≈80%) reported using the Internet in the past year, leaving a sizable minority offline or under-connected. These rich, nationally representative microdata—covering home technology (computers, smartphones, etc.), connectivity, and usage practices—now allow researchers to move beyond simple averages to profile which subpopulations are left behind (INEC, 2025).

Based on this framework, the present study is guided by the following research questions: (RQ1) What is the current extent of digital inequality in Ecuador in terms of ICT access, device ownership, and internet use? (RQ2) To what extent are age, educational attainment, and area of residence associated with disparities in ICT access and use? (RQ3) What multidimensional profiles of digital inclusion and exclusion emerge when these ICT indicators and sociodemographic characteristics are jointly analyzed using Multiple Correspondence Analysis (MCA)?

While the first two research questions are addressed through descriptive and associative analyses aimed at documenting the magnitude and structure of inequalities, the third question adopts an exploratory perspective, using MCA to identify latent patterns and profiles of digital inequality. In this sense, the study combines a diagnostic approach with an exploratory multivariate analysis, contributing a synthetic and empirically grounded assessment of digital inequality in Ecuador.

This study contributes to the literature on digital inequality in Ecuador and Latin America in three main ways. First, it provides an updated, nationally representative assessment based on ENEMDU 2025 microdata, capturing digital disparities in a context of expanded connectivity and ongoing digital transformation policies. Second, by integrating household- and individual-level indicators, the analysis offers a comprehensive view of the digital divide that goes beyond single-level approaches. Third, the use of Multiple Correspondence Analysis (MCA) enables the identification of multidimensional profiles of digital inclusion and exclusion shaped by the interaction of age, education, and territory, adding analytical depth to previous descriptive studies. By situating Ecuador within comparative debates on digital stratification in middle-income countries, the study also contributes to the broader international discussion on how expanding connectivity interacts with persistent structural inequalities.

## Materials and methods

### Design and participants

This study employed a cross-sectional, observational, and analytical design based on secondary data from the National Survey of Employment, Unemployment, and Underemployment (ENEMDU) conducted by the National Institute of Statistics and Censuses (INEC) of Ecuador in July 2025 (Hunziker and Blankenagel, 2024). ENEMDU is a nationally representative household survey integrated into the System of Integrated Household Surveys and uses a probabilistic, stratified, and multistage sampling design (INEC, 2025). The ICT module covers households and individuals aged 5 years and older, with specific indicators for digital literacy assessed among individuals aged 15–49 years. For the present analysis, both household-level and individual-level records were considered, applying the official expansion factors provided by INEC to ensure national representativeness.

### Instrument and data collection

The study used the ICT module of ENEMDU 2025, which collects information on household technological equipment (desktop computers, laptops, and tablets), internet access, internet use, mobile phone ownership, smartphone use, and digital illiteracy. Data were collected through face-to-face interviews using a standardized questionnaire administered by trained enumerators, following INEC's methodological guidelines. The module has been implemented consistently since 2022, allowing for methodological stability and comparability across recent years (INEC, 2025).

### Ethical considerations

This research analyzed publicly available, anonymized secondary data provided by INEC (Zuo et al., 2021). No personal identifiers were included in the datasets. In accordance with international ethical standards for research using secondary data, informed consent was obtained by INEC during the original data collection process. The present study did not require additional ethical approval, as it involved no direct interaction with participants and posed minimal risk (Chatfield and Dove, 2023). The present analysis complied with international ethical guidelines for research using secondary, anonymized survey data.

### Statistical analysis

Data analysis was conducted using Python in the Google Colab environment. Descriptive statistics were first computed to characterize ICT access and use (Lybeck et al., 2024). All statistical analyses were conducted using Python (version 3.10) on the Google Colab platform. Data management and descriptive analyses were performed using pandas (v1.5.3) and numpy (v1.23). Bivariate analyses were conducted with scipy (v1.9). The Multiple Correspondence Analysis was implemented using the prince library (v0.12.0), and graphical outputs were generated using matplotlib (v3.7) and seaborn (v0.12). Subsequently, Multiple Correspondence Analysis (MCA) was applied to explore multidimensional patterns of digital inequality across sociodemographic variables. All analyses accounted for survey weights to preserve population-level inference (Atkinson, 2024). Survey weights were applied in the univariate and bivariate analyses to ensure national representativeness. For the MCA, weights were not directly incorporated into the computation of factorial axes, in line with common practice in exploratory multivariate analysis and acknowledging ongoing methodological debates regarding the use of sampling weights in MCA. Accordingly, the MCA results are interpreted as exploratory patterns of association within the sample structure, while population-level inferences rely primarily on the weighted descriptive and bivariate results.

MCA was implemented as an exploratory technique to map relational structures among categorical modalities. The first two dimensions were interpreted using category coordinates and their contributions to inertia, prioritizing sociological interpretability over variance maximization. Dimension labels were assigned based on the clustering of modalities across age, education, territory, and ICT indicators, consistent with multi-level digital divide theory. Graphical outputs include (i) a category map to visualize modality proximities and (ii) an individuals map stratified by area to examine territorial differentiation in profile distributions. All sociodemographic and ICT variables included in the MCA were treated as active variables in the construction of factorial axes. No supplementary variables were projected *post hoc*. This decision ensures that the extracted dimensions reflect the joint structure of access, use, and structural stratifiers, rather than privileging any single domain.

Variable operationalization followed INEC ICT-module definitions. Age was analyzed using policy-relevant life-course groupings (5–14, 15–24, 25–34, 35–44, 45–54, 55–64, and 65+), education was grouped into interpretable attainment categories aligned with Ecuador's educational structure, and ICT indicators were recoded as categorical modalities representing access (household internet), device availability (computer, smartphone), and use (individual internet use). Records with missing responses in the active MCA variables were excluded listwise for the MCA step to preserve category comparability, while weighted descriptive and bivariate estimates retained valid denominators per indicator.

The selection of variables included in the Multiple Correspondence Analysis (MCA) followed an explicit analytical strategy grounded in the multi-level digital divide framework. Variables were chosen to represent key dimensions of digital inequality consistently highlighted in the literature, including ICT access and device availability (first-level digital divide), internet use and basic digital engagement (second-level digital divide), and core sociodemographic stratifiers (age group, educational attainment, and area of residence). While the availability of standardized indicators in the ENEMDU 2025 ICT module enabled this selection, the inclusion criteria were theory-driven rather than purely data-driven.

Variable selection and conceptual framing align with established frameworks that operationalize digital inequality's structural, cognitive, and motivational dimensions (Ragnedda et al., 2022; Scheerder et al., 2017; Szabó, 2024). The resulting MCA dimensions should be understood as reflecting patterns of digital inclusion and exclusion within the scope of these analytically defined indicators, rather than as an exhaustive representation of all possible dimensions of digital inequality. Consequently, the MCA results are interpreted as exploratory mappings of relative positions and profiles, not as causal relationships or comprehensive measures of digital disadvantage.

The statistical analysis comprised a sequential strategy combining descriptive, inferential, and multivariate techniques. First, univariate descriptive statistics were computed to summarize the distribution of ICT access, device ownership, and internet use at both household and individual levels (Scott Jones, 2022). Second, bivariate analyses were conducted using the chi-square (χ^2^) test of independence to examine associations between ICT indicators and sociodemographic variables (age, sex, area of residence, and education; Chicco et al., 2025; Wuensch, 2025). The strength of these associations was quantified using Cramér's V, with conventional thresholds applied to interpret weak, moderate, and strong relationships (Nwaigwe and Wobo, 2024). All bivariate analyses were performed using survey-weighted frequencies to preserve national representativeness. Finally, a Multiple Correspondence Analysis (MCA) was applied to explore multidimensional patterns of digital inequality and to identify latent profiles of digital inclusion and exclusion across combined ICT and sociodemographic categories. Statistical significance was assessed at a 5% level (*p* < 0.05). Sensitivity checks were conducted by verifying that the general structure of associations remained stable when excluding individual variables from the MCA, confirming the robustness of the identified relational patterns.

The selection of variables included in the Multiple Correspondence Analysis (MCA) was guided by an explicit analytical framework derived from the literature on the multi-level digital divide. Specifically, variables capturing ICT access and device ownership (first-level digital divide), internet use and basic digital engagement (second-level digital divide), and key sociodemographic stratifiers (age group, educational attainment, and area of residence) were included due to their consistent relevance in prior empirical research. This theory-driven approach ensured that the MCA focused on analytically meaningful dimensions of digital inequality rather than being determined solely by data availability.

## Results

The univariate analysis reveals that ICT access in Ecuador is widespread but highly unequal. In July 2025, 71.3% of households had internet access, equivalent to approximately 3.75 million households, while 28.7% (1.5 million) remained disconnected. A pronounced urban–rural gap persisted, with internet access reaching 76.6% in urban areas compared to 58.7% in rural areas, a difference of 17.9 percentage points. Internet use at the individual level was relatively high **(**82.4%), indicating that connectivity does not fully translate into advanced digital engagement. Household ICT profiles showed a strong dependence on mobile technologies, with smartphones present in 91.3% of households, whereas computers were available in only 41.0%. These findings highlight a multidimensional digital inequality, characterized not only by access gaps but also by limitations in devices and digital competencies, with important implications for inclusive digital policies (Table 1).

**Table 1:** Key univariate indicators of ICT access and use in Ecuador (ENEMDU 2025).

Indicator	Value (%)
Households with internet access	71.3 (3.75 million)
Internet access—urban areas	76.6
Internet access—rural areas	58.7
Individuals using the internet	82.4
Households with smartphones	91.3
Households with computers	41.0
Individuals with an active mobile phone	61.9

The bivariate analysis confirms that digital inequality in Ecuador is primarily structured by age and place of residence. Age shows the strongest association with internet use (Cramér's *V* = 0.4517; *p* < 0.001), revealing a pronounced generational divide: internet use reaches 98.7% among individuals aged 15–24, but drops sharply to 45.2% among those aged 65 and older. Geographic location is also a significant determinant. Internet access is higher in urban areas (76.6%) than in rural areas (58.7%), with a moderate association (*V* = 0.1851; *p* < 0.001). Similarly, computer ownership varies substantially by area (urban: 38.5% vs. rural: 15.9%), showing a moderate association (*V* = 0.2390). In contrast, sex is not a significant factor in mobile phone ownership (*V* = 0.0122; *p* = 0.0565), indicating near gender parity. Overall, the results demonstrate that Ecuador's digital divide is multidimensional, driven mainly by generational and territorial inequalities rather than gender differences, with clear implications for targeted public policies (Table 2).

**Table 2:** Summary of bivariate associations between ICT indicators and sociodemographic factors.

Relationship	Cramér's *V*	*p*-value	Strength	Interpretation
Internet use × age	0.4517	<0.001	Strong	Clear generational digital divide
Computer ownership × area	0.2390	<0.001	Moderate	Infrastructure gap urban–rural
Internet access × area	0.1851	<0.001	Moderate	Persistent territorial inequality
Mobile phone × sex	0.0122	0.0565	Weak	No significant gender gap

**Figure 1:**
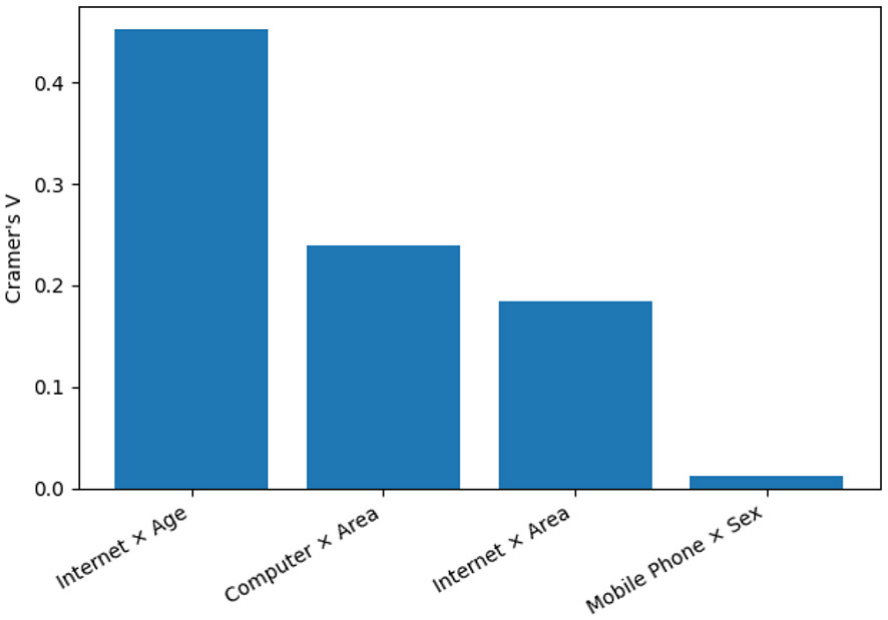
Strength of associations (Cramér's *V*) between ICT indicators (internet use, internet access, computer ownership, and mobile phone ownership) and sociodemographic factors (age group, area of residence, and sex), based on weighted ENEMDU 2025 data.

The bar chart above illustrates the relative magnitude of associations (Cramér's *V*), highlighting age as the dominant factor shaping digital inequality, followed by geographic area, while sex shows a negligible effect (Figure 1).

**Figure 2:**
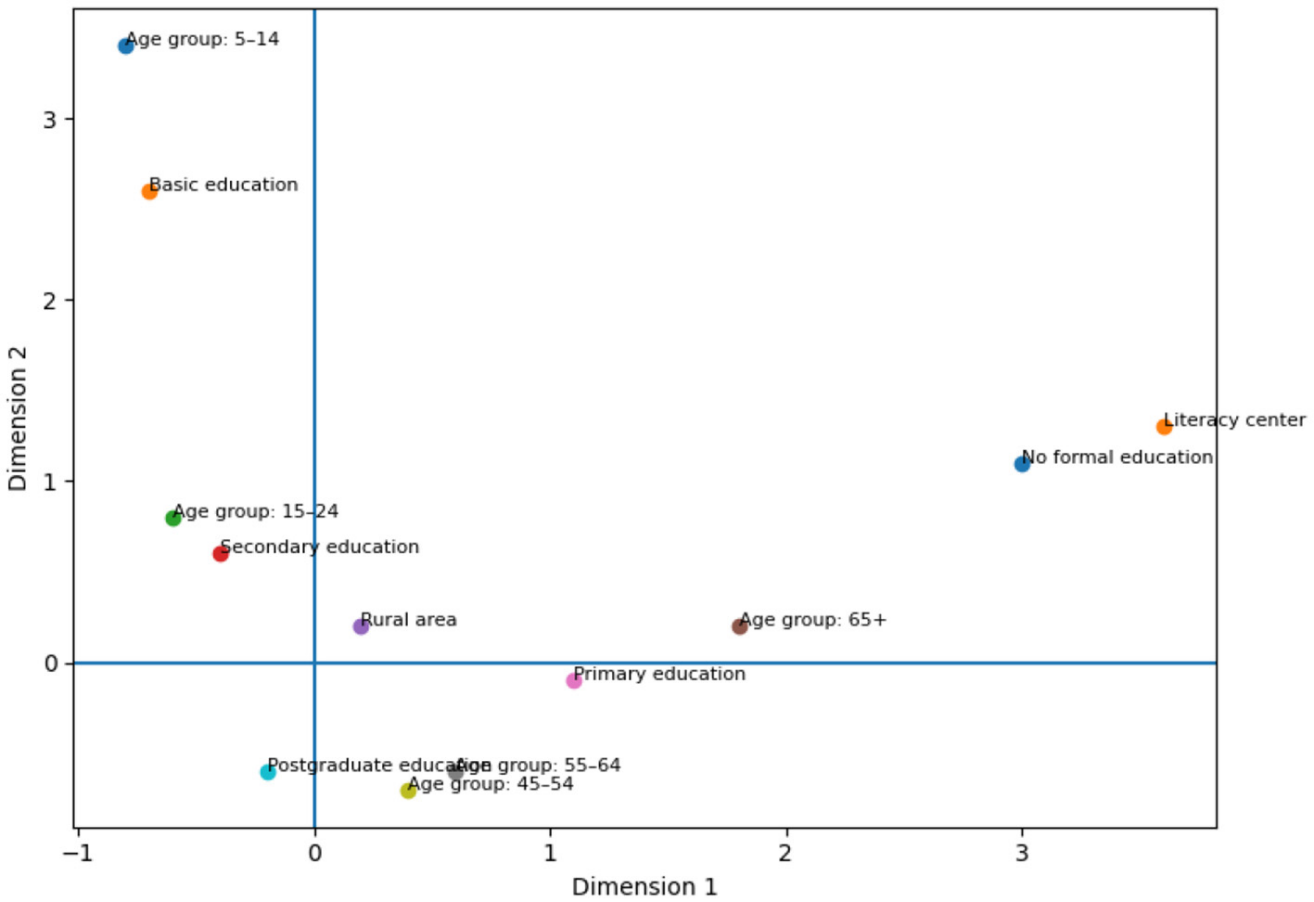
Multiple correspondence analysis (MCA) category map showing the relative positioning of ICT access and use indicators, age groups, educational attainment levels, and area of residence (urban/rural) in a two-dimensional factorial space (Dimensions 1 and 2), based on ENEMDU 2025 data.

*The Multiple Correspondence Analysis (MCA) provided an integrated multivariate* representation of digital inequality in Ecuador by jointly examining ICT access, device availability, and sociodemographic characteristics using ENEMDU 2025 data. The first two dimensions explained 21.20% of total inertia (Dimension 1: 12.52%; Dimension 2: 8.68%), which is acceptable for exploratory analyses involving multiple categorical indicators. Dimension 1 represents a digital inclusion–exclusion gradient, while Dimension 2 captures a demographic–territorial contrast, mainly driven by age groups and area of residence. Although this proportion may appear modest, it is consistent with expectations for large-scale social survey data characterized by high categorical diversity. In line with established methodological literature, the interpretative value of MCA in this context lies less in maximizing explained variance and more in identifying meaningful relational structures among categories (Figure 2).

**Figure 3:**
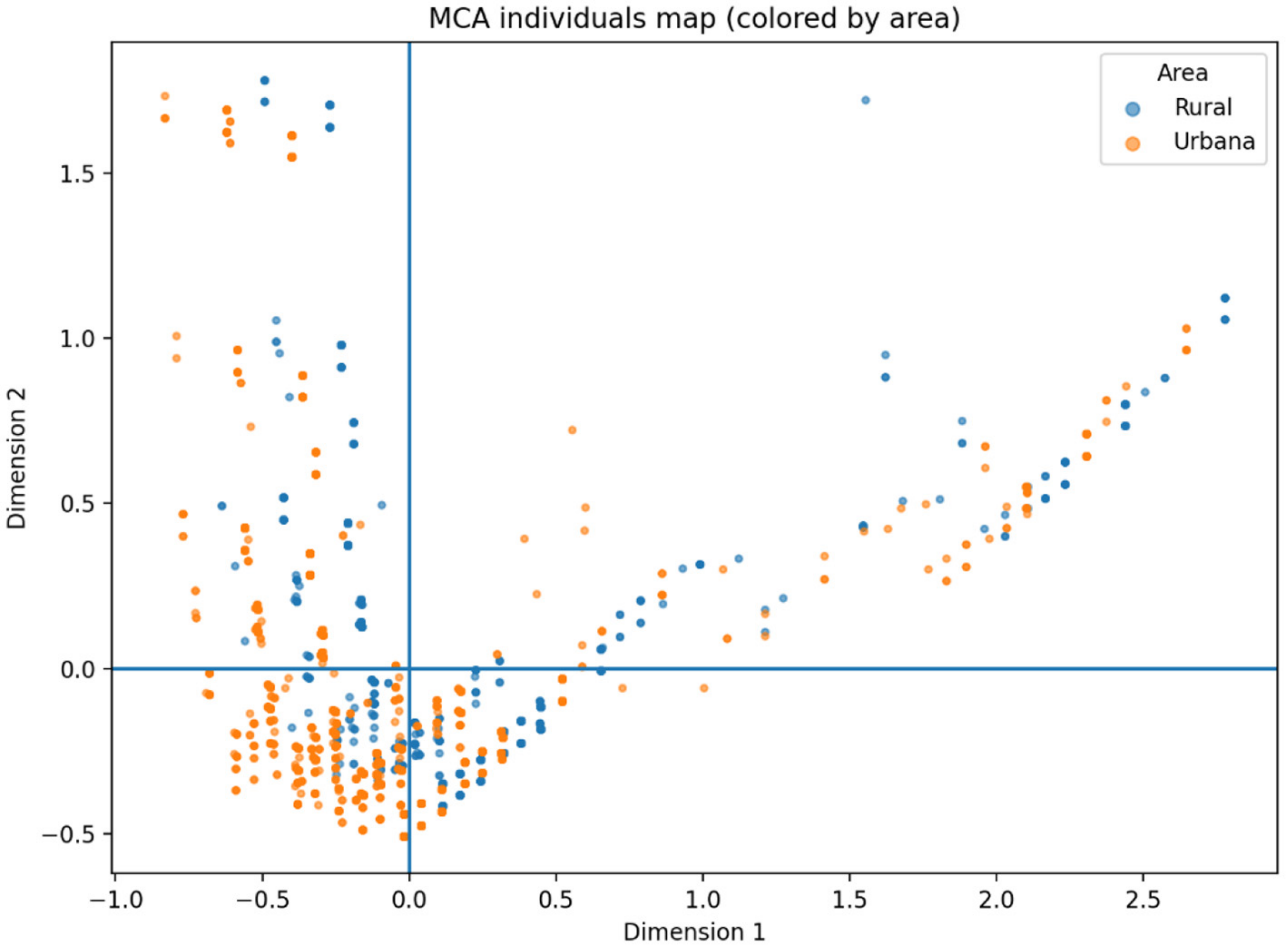
MCA individuals map displaying the distribution of respondents in the factorial space (Dimensions 1 and 2), colored by area of residence (urban vs. rural), illustrating territorial patterns of digital inclusion and exclusion based on ENEMDU 2025 data.

Figure 3 displays the distribution of individuals in the two-dimensional factorial space derived from the MCA, stratified by area of residence. The visual pattern indicates a clearer concentration of rural respondents along the pole associated with lower digital access and limited device diversification, whereas urban respondents are more frequently positioned along the axis linked to higher educational attainment and diversified ICT use. This spatial differentiation reinforces the territorial dimension of digital inequality identified in the weighted descriptive and bivariate analyses.

To complement the exploratory MCA, a stratified analysis (age × area) examined weighted proportions of internet use. Results show that age-related inequalities are amplified by place of residence, with near-universal use among youth (15–34 years) in both areas, but sharp declines among older adults—particularly ≥65 years in rural areas.

While many of the observed inequalities are consistent with previous regional and international studies, this analysis provides several new insights. First, the use of recent ENEMDU 2025 data allows for an updated assessment of digital inequality in a context of expanded connectivity and ongoing digital transformation policies. Second, the multivariate MCA approach reveals how age, education, and area of residence combine to form distinct profiles of digital inclusion and exclusion, rather than operating as isolated determinants. This profile-based perspective adds nuance to existing evidence by highlighting cumulative and intersecting disadvantages that are not fully captured in earlier unidimensional analyses.

## Discussion

Rather than introducing new policy frameworks, the contribution of this study lies in providing updated, nationally representative empirical evidence that confirms the persistence of well-documented digital inequalities. The use of ENEMDU 2025 data allows for timely monitoring of whether current digital inclusion strategies are effectively reaching older adults and rural populations, thereby supporting evidence-based policy adjustment rather than policy redesign.

The patterns identified in Ecuador are consistent with recent international evidence demonstrating that age and territorial location remain persistent determinants of digital inequality across diverse socio-economic contexts. Comparative research in Europe and North America has documented enduring generational divides in digital skills, online engagement, and diversity of use, even in settings with high connectivity penetration (OECD, 2023; Van Deursen and Helsper, 2015). Likewise, global reports from the International Telecommunication Union (ITU, 2023) emphasize that rural–urban disparities in digital infrastructure and service quality remain structurally embedded across both high- and middle-income countries. In this regard, the Ecuadorian case reflects broader global patterns rather than an isolated national phenomenon. Similar multidimensional configurations have been reported in recent studies of Southern Europe and emerging economies, where digital inequalities increasingly shift from connectivity gaps to disparities in device diversification and digital returns (Bergantino et al., 2025; de Clercq et al., 2023). Beyond confirming well-documented patterns of digital inequality, the added value of this study lies in its ability to empirically consolidate and update existing evidence using recent nationally representative data, while revealing cumulative and profile-based dimensions of exclusion through a multivariate exploratory approach.

This study provides descriptive and exploratory evidence that digital inequality in Ecuador remains multidimensional, structural, and intersectional, despite relatively high levels of aggregate ICT penetration reported in recent years. By integrating univariate, bivariate, and multivariate analyses using ENEMDU 2025 data, the findings extend prior descriptive work and contribute a more nuanced understanding of how age, territory, and access to devices jointly shape digital inclusion, aligning with contemporary theoretical frameworks of the digital divide (Ragnedda et al., 2022).

At the descriptive level, the results confirm that while internet access (71.3%) and individual use (82.4%) are widespread, substantial gaps persist, particularly between urban and rural households. This pattern mirrors regional evidence reported for Latin America and the Caribbean, where territorial disparities continue to constrain equitable digital development (ECLAC, 2024). The strong reliance on mobile technologies, contrasted with the low penetration of computers, suggests that connectivity alone does not guarantee access to higher-order digital skills related to education, employment, and productivity. This supports the notion of a second-level digital divide, where differences in devices and competencies become as relevant as access itself (Ragnedda et al., 2022).

Bivariate results highlight age as the most influential determinant of digital inequality, with a strong association between age and internet use. The pronounced gap between young adults (15–24 years) and older adults (≥65 years) is consistent with international evidence indicating that generational factors strongly condition digital engagement (Moreno-Vallejo et al., 2025). Importantly, sex was not a significant determinant of mobile phone ownership, suggesting that gender parity has largely been achieved in basic access, at least for mobile connectivity. This finding refines earlier assumptions of gender-based digital exclusion and indicates that policy efforts in Ecuador may now be better directed toward other vulnerable groups.

The Multiple Correspondence Analysis (MCA) adds critical insight by revealing how multiple disadvantages accumulate. Dimension 1 clearly represents a digital inclusion–exclusion gradient, while Dimension 2 reflects a demographic–territorial axis, confirming that digital inequality cannot be explained by single variables in isolation. The stratified analysis further demonstrates that age-related disparities are amplified in rural contexts, particularly among older adults, indicating compounded vulnerability. These findings resonate with recent Ecuadorian and international studies emphasizing that digital exclusion often emerges from the interaction of social, economic, and spatial factors rather than from isolated deficits (De la Cruz Campos et al., 2024).

Importantly, the MCA patterns can be interpreted as evidence of structurally layered digital inequality rather than isolated gaps. In line with international digital stratification research, Ecuador's profiles suggest a cumulative disadvantage mechanism: lower education, rural residence, and older age converge with lower device diversification and narrower forms of internet engagement, limiting opportunities to translate connectivity into tangible socio-economic returns. This matters because “mobile-first” access—while widespread—often supports social and entertainment uses more than productive and skills-intensive activities, reinforcing second- and third-level digital divides. Thus, the Ecuadorian case reflects a broader global pattern in which inequalities shift from connectivity to capabilities, quality of use, and outcomes, especially for older adults and territorially marginalized populations.

Beyond issues of access, international scholarship increasingly highlights second- and third-level digital divides, referring to disparities in digital skills, quality of use, and the socio-economic returns derived from online participation (Van Deursen and Helsper, 2015). The multidimensional profiles identified through the MCA align with this perspective, as they reveal differentiated patterns of digital engagement that extend beyond simple connectivity. In particular, the distinction between mobile-dependent usage and diversified digital practices reflects broader global debates on digital capital accumulation and unequal capacity to convert digital access into social and economic advantages.

Comparatively, the strong age gradient observed in Ecuador mirrors findings reported across multiple contexts, where older cohorts systematically show lower digital participation and lower digital returns even when infrastructure improves. Likewise, the persistent rural–urban structure in internet access and computer availability resembles international evidence emphasizing that territorial inequalities remain durable because they are linked to broader infrastructure, service quality, and affordability constraints. In this sense, Ecuador should be interpreted as a confirmatory but policy-relevant case: it illustrates how middle-income countries can reach relatively high aggregate connectivity while still reproducing global digital inequality dynamics through stratified access to devices, skills, and meaningful use. This generational gradient is widely observed in cross-national analyses, where older cohorts consistently demonstrate lower digital engagement and weaker digital returns even under expanding connectivity conditions (Hollimon et al., 2025; OECD, 2023).

From a sociological perspective, the first MCA dimension can be interpreted as reflecting cumulative advantage and disadvantage in digital inclusion. Categories associated with younger age groups, higher educational attainment, urban residence, and access to multiple ICT devices cluster on the positive pole, consistent with theories of stratification and resource accumulation. Conversely, the negative pole groups older age, lower education, and rural residence, illustrating how digital exclusion aligns with broader patterns of social inequality.

The second dimension captures heterogeneity in modes of digital engagement rather than simple access. This axis differentiates profiles characterized by mobile-only connectivity and basic internet use from those associated with more diversified and productive digital practices. This pattern resonates with second-level digital divide theory, which emphasizes inequalities in usage intensity and quality beyond mere connectivity.

Thus, the dimension labels are grounded in established sociological frameworks of digital stratification rather than serving as purely descriptive summaries of the data structure.

From a policy perspective, the results suggest that universal connectivity strategies are necessary but insufficient. While infrastructure expansion remains crucial—especially in rural areas—targeted interventions are needed to address digital skills among older adults and to expand access to productivity-enhancing devices such as computers. Programs aligned with Ecuador's Digital Transformation Agenda 2025–2030 should therefore adopt a differentiated approach, prioritizing populations facing overlapping forms of exclusion.

Overall, this study demonstrates the analytical value of combining MCA with stratified analyses to inform evidence-based digital policies. By leveraging nationally representative microdata, it advances the understanding of digital inequality in Ecuador and provides a methodological framework applicable to other middle-income countries facing similar challenges.

Given the exploratory nature of Multiple Correspondence Analysis, the patterns identified in this study should be interpreted as descriptive and indicative rather than causal. The results provide an empirically grounded mapping of multidimensional profiles of digital inclusion and exclusion, which can inform, but not by themselves determine, policy design. Consequently, the policy implications discussed should be understood as suggestive directions for targeted interventions and as a basis for future longitudinal or causal analyses.

From a broader sociological standpoint, these findings support theories of cumulative disadvantage and digital stratification, which suggest that technological inequalities are intertwined with pre-existing social hierarchies (Ragnedda et al., 2022). Rather than representing a temporary technological lag, digital exclusion appears embedded within structural inequalities in education, territory, and life-course trajectories. The Ecuadorian evidence thus contributes to global discussions on how digital transformation may reproduce or intensify existing forms of socio-economic stratification.

Therefore, the contribution of this study lies not in proposing new theoretical frameworks or policy instruments, but in empirically consolidating and updating existing knowledge through nationally representative and methodologically transparent analysis. This strengthens the evidence base for monitoring and prioritizing digital inclusion efforts in Ecuador and comparable middle-income contexts.

## Limitations

The study is based on a cross-sectional design, which limits the ability to establish causal relationships between sociodemographic factors and ICT access or use. Although the ENEMDU 2025 provides high-quality, nationally representative data, the observed associations reflect patterns at a single point in time and do not capture dynamic changes in digital inclusion trajectories.

While Multiple Correspondence Analysis (MCA) is well suited for exploring complex categorical relationships, it is inherently exploratory. The interpretation of dimensions depends on the selected variables and categories, and the proportion of explained inertia, although acceptable for social survey data, indicates that additional unobserved factors may also contribute to digital inequality in Ecuador.

In addition, the analysis does not explicitly account for certain variables that may be endogenously related to digital access and use, such as household income or the quality and stability of internet connections. While these factors are partly correlated with education and area of residence, their omission may limit the explanatory scope of the results and should be addressed in future research.

Additionally, the absence of longitudinal data limits the ability to assess trajectories of digital inclusion over time, which would be particularly relevant in contexts of rapid technological expansion.

## Conclusion

The policy conclusions derived from this study should be interpreted within the limits of its data and methods. Given the cross-sectional design and the exploratory nature of the analytical approach, the findings do not aim to propose new policy instruments or causal interventions. Rather, they provide updated and nationally representative empirical confirmation of well-documented patterns of digital inequality.

Digital inequality in Ecuador is multidimensional and structurally rooted. Despite relatively high levels of internet access and use, substantial disparities persist, particularly across age groups and geographic areas. The results demonstrate that digital exclusion is not solely a matter of connectivity, but also of access to devices and digital competencies.

Age and territory are the primary drivers of digital exclusion. Generational gaps, especially affecting adults aged 65 years and older, are markedly intensified in rural areas. In contrast, gender differences in basic mobile access are negligible, suggesting that policy priorities should shift toward addressing territorial and generational vulnerabilities.

Multivariate approaches enhance evidence-based digital policymaking. The combination of MCA and stratified analyses proved effective in identifying complex profiles of digital inequality. These findings support the need for targeted and differentiated public policies, focusing on rural infrastructure, digital skills training for older adults, and expanded access to productivity-enhancing technologies such as computers, in line with Ecuador's Digital Transformation Agenda.

## Data Availability

Publicly available datasets were analyzed in this study. This data can be found here: https://www.ecuadorencifras.gob.ec/tecnologias-de-la-informacion-y-comunicacion-tic/.
